# Physiological and functional characterization for high‐throughput optogenetic skeletal muscle exercise assays

**DOI:** 10.1002/btm2.70101

**Published:** 2025-12-14

**Authors:** Ronald H. Heisser, Angel Bu, Laura Schwendeman, Tamara Rossy, Pavankumar Umashankar, Vincent Butty, Ritu Raman

**Affiliations:** ^1^ Department of Mechanical Engineering Massachusetts Institute of Technology Cambridge Massachusetts USA; ^2^ Koch Institute for Integrative Cancer Research Massachusetts Institute of Technology Cambridge Massachusetts USA

**Keywords:** exercise, optogenetics, skeletal muscle, tissue engineering

## Abstract

Exercise promotes human mobility by tuning the function of skeletal muscle, and recent studies highlight exercise's broader impacts on human health via muscle's paracrine and endocrine roles beyond force generation. In vitro models of tissue engineered skeletal muscle enable precise investigation of adaptation to exercise, with emerging approaches for optogenetic muscle stimulation providing a less invasive alternative to traditional techniques for electrical stimulation. In this study, we present a high‐throughput muscle culture and optical exercise protocol for scalable in vitro exercise studies. First, we characterize optical rheobase for 2D muscle monolayers, finding that optical intensities as low as 5 μW mm^−2^ can trigger functional contraction. We then leverage RNA sequencing to map changes in muscle gene expression in response to various optical exercise regimens, highlighting how changing stimulation parameters impact myogenic and broader physiological and pathological transcriptional responses. Our platform and results establish a practical foundation for high‐throughput in vitro exercise studies of skeletal muscle.

**Translational Impact Statement**

Robust in vitro models of tissue engineered skeletal muscle enable investigating exercise‐mediated responses at the cell and tissue level. We leverage optogenetic techniques and open‐source hardware to develop a minimally invasive high‐throughput muscle exercise assay that avoids common pitfalls of electrical stimulation including media electrolysis and complicated experimental procedures. Our study generates RNA sequencing datasets in response to several muscle training regimens (spanning a range of stimulation frequencies and durations), revealing physiological and pathological responses to exercise and informing practical next steps for cultivating more mature skeletal muscle models.

## 1 INTRODUCTION

Mobility is a critical indicator of longevity in human health,^1^, ^2^, ^3^ and exercise can sustain a level of skeletal muscle composition necessary for bodily resilience (such as impact from falls), strength, and cardiovascular health.^4^ Beyond a biomechanical role, recent research has implicated muscle contraction in regulating biochemical communication with many organ systems. While glucose uptake and thermogenic pathways have been understood for decades, recent studies have highlighted that exercising muscles also release signaling molecules, “myokines,” that influence vascularization, bone formation, immune system function, peripheral nerve growth, and even glucagon‐like peptide‐1 (GLP‐1) secretion.^5^, ^6^, ^7^, ^8^

Tissue engineering can help advance understanding of muscle physiology by enabling high‐throughput in vitro observation of changes in muscle fiber morphology, contractility, and gene expression.^9^, ^10^ Reproducible protocols for culturing and exercising skeletal muscle tissues in vitro can address current drawbacks of in vivo studies, in which exercise regimens can be difficult to uniformly apply across individuals, and muscle‐specific responses can be hard to isolate.^11^, ^12^

Most in vitro exercise studies have leveraged electrical stimulation to trigger repeated muscle contraction. In this approach, applying an electric field to cell culture media opens voltage‐gated ion channels in the cell membrane, including the voltage‐gated calcium ion channels that trigger excitation‐contraction coupling. Studies have reported that prolonged electrical stimulation of engineered muscle tissues leads to deterioration of contractile performance over time, potentially due to electrolysis of the culture media that can change pH.^13^, ^14^, ^15^, ^16^ Moreover, electrical stimulation typically generates bubbles that reduce optical accessibility and the ability to record precise functional readouts via optical microscopy. Finally, electrical stimulation requires placing tissues between pairs of precisely spaced electrodes, often requiring extensive manual handling steps and multi‐step electrode sterilization protocols.

Advances in optogenetics have enabled a less invasive alternative to electrical stimulation of skeletal muscle. In this approach, muscle cells can be engineered to express light‐gated membrane calcium ion channels that can be specifically activated to initiate contraction. Optical stimulation also offers precise spatial control and could be used to pattern parts of a tissue, rather than the tissue as a whole, aligning with in vivo studies that highlight the benefits of spatially targeted muscle stimulation.^17^, ^18^ A current drawback of optogenetic exercise approaches is that the lack of commercially available optogenetic cell lines limits broader access. However, several papers have reported effective methods for researchers to engineer optogenetic muscle cells and also highlighted best practices for avoiding off‐target effects of genetic engineering on downstream muscle contractility.^19^, ^20^

In previous studies, we have leveraged optical stimulation to exercise tissue‐engineered 3D muscle constructs in vitro and in vivo, and demonstrated that daily optical stimulation can increase contractile force by ~300%.^18^, ^21^ However, these studies did not systematically investigate or optimize how different optical exercise training regimens impact muscle gene expression. Moreover, given the opacity of 3D muscle tissues, such culture platforms do not enable uniformly stimulating all cells within a tissue at the same light intensity. Precise investigation of muscle transcriptional responses to prolonged optical stimulation thus requires scalable methods for culturing and uniformly optically stimulating 2D monolayers of contractile muscle.^22^, ^23^, ^24^, ^25^, ^26^

Historically, 2D skeletal muscle cultures have rarely been leveraged for exercise studies, as muscle monolayers typically delaminate from underlying substrates within a few days of culture due to generated passive tension and active contractile forces.^27^ To address this challenge, we have recently demonstrated that differentiating 2D skeletal muscle monolayers on micro‐topographically patterned hydrogels enables longitudinal studies of aligned contractile tissues over several weeks.^7^, ^28^ In this study, we leverage this tissue fabrication protocol and combine it with open‐source hardware for optical stimulation of standard multi‐well tissue culture plates.^29^, ^30^ We first establish minimum intensity thresholds, or rheobase, for inducing twitch in optogenetic skeletal muscle fibers derived from the widely used C2C12 line expressing the blue light sensitive calcium ion channel ChR2(H134R).^13^, ^17^, ^21^, ^23^, ^26^, ^31^, ^32^, ^33^, ^34^, ^35^, ^36^ To our knowledge, optical rheobase is not currently documented in the optogenetic muscle literature. We then conduct high‐throughput optical exercise experiments of varying stimulation frequency (1–10 Hz) and duration (0–60 min) and leverage bulk RNA sequencing (RNA‐seq) to examine how modulating training regimen impacts muscle physiological and pathological gene expression. Our findings establish the diverse effects of optically exercising muscle tissues, with the goal of establishing a practical foundation for researchers to leverage optogenetics to promote maturation of tissue engineered skeletal muscle.

## 2 RESULTS

### 2.1 Optical excitation‐contraction characterization

**FIGURE 1:**
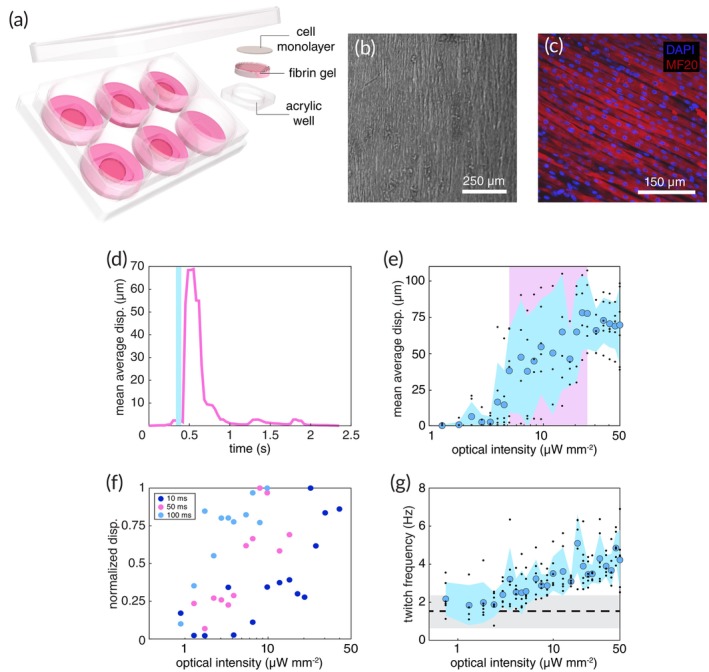
Optical excitation‐contraction coupling characterization. Six biological replicates were cultured and characterized for illumination sensitivity and twitch displacement. (a) Cells were seeded on fibrin hydrogels containing 25 μm grooves. Hydrogels were cast within 15 mm diameter acrylic wells within standard six‐well plates, enabling even illumination of tissues with a 470 nm light source. (b) Brightfield microscope image of aligned muscle fibers on day 12 of culture in differentiation medium (DM), without exercise. (c) Fluorescence microscope image showing muscle nuclei (DAPI, blue) fusing to form multinucleated fibers expressing myosin (MF20, red). (d) Contractile response of a representative muscle tissue in response to a 470 nm light pulse with an intensity of 25 μW mm^−2^. The blue band shows the duration of the optical pulse. (e) Muscle displacement in response to optical stimulation at varying intensities for six biological replicates. Blue points show average values for each measured intensity. Black dots show individual measurements taken across all samples. The pink shaded region shows the range of individual rheobases measured across all replicates. Blue shaded region shows standard deviation of measurements. (f) Normalized optical response of a single muscle tissue exposed to varying optical pulse widths, showing a trend of lower rheobase for longer optical pulses. (g) Average twitch frequency of six biological replicates under continuous illumination. Blue points show average values for each measured intensity. Black points show individual measurements taken across all samples. Dotted line and gray shading correspond to average and standard deviation of spontaneous twitch recorded without optical stimulation. Shaded blue region gives standard deviation of each twitch frequency measurement.

C2C12 myoblasts expressing the blue light‐sensitive calcium ion channel ChR2(H134R) were differentiated into 2D muscle sheets on fibrin hydrogels containing 25 μm parallel grooves, using established protocols (Figure 1a–c).^7^, ^17^, ^21^, ^28^ Tissues were fabricated and maintained within a 15 mm diameter acrylic well (similar to the size of a 24‐well plate) within a standard 6‐well plate to enable evenly illuminating the entire 2D tissue surface with a 470 nm optical fiber connected to a collimating adaptor (Figure S1, Supporting Information). We observed spontaneous contractions in all samples by day 6 of DM culture, and the characterization study was conducted on day 9.

We obtained optical excitation‐contraction response curves for six biological replicates by testing 50 ms light pulse durations with intensities between 0.75 and 50 μW mm^−2^. Fifty‐millisecond pulse widths have been used in previous optogenetic exercise studies and help to distinguish stimulated contractions from spontaneous contractions at lower intensity ranges.^26^ Videos of muscle contraction were recorded at 30 fps with a microscope camera, and open‐source computational frameworks^37^, ^38^ were used to find peak displacement amplitudes for each tissue (Figure 1d, Video S1). We observed that optically stimulated contractions became perceptibly distinct from spontaneous contractions at intensities as low as 3 μW mm^−2^, and that all tissues responded to stimulation at thresholds below 20 μW mm^−2^. Figure 1e shows an increasing contractile response that, on average, plateaus around 25 μW mm^−2^. This intensity range is lower than reported in previous in vitro experiments with 2D optogenetic muscle, which use light intensities similar to those used during in vivo experiments (0.1–1 mW mm^−2^).^22^, ^23^, ^24^, ^39^ These experiments suggest that the minimal light intensity required to activate contraction, or rheobase, in 2D C2C12‐derived skeletal muscle tissues stimulated at 50 ms pulse durations is in the range of 5–25 μW mm^−2^. Figure 1f compares contractile responses for variable pulse widths, showing the tradeoff between threshold stimulation intensity and pulse width. Selecting the optimal twitch stimulation parameters likely involves balancing optical dose (the product of intensity and pulse width, given in nJ) and aggregate channel opening dynamics. Figure 1f highlights that our minimum optical dose ranged from 200 to 300 nJ, in agreement with reported electrical pulse energy values from an earlier rheobase characterization study.^15^ In vivo experiments have previously shown that continuous (rather than pulsed) optical illumination causes a tetanic response that decays back to a relaxed state in seconds.^40^ In contrast, we observed that varying light intensity influenced the frequency of spontaneous twitch in the tissues, with sustained illumination increasing twitch frequency from about 2 to 4 Hz (Figure 1g, Video S2).

Defining rheobase in our platform enabled determination of the minimum optical dose required to trigger muscle contraction. Leveraging these findings, we studied the impacts of various optical stimulation frequencies (1–10 Hz) and durations (0–60 min) on muscle gene expression. While we focused on tissue responses to exercise training, this platform can also be used to study cellular responses to optical exposure at varying intensities, including phototoxic effects as discussed later in the results section.

### 2.2 High‐throughput exercise experiment

**FIGURE 2:**
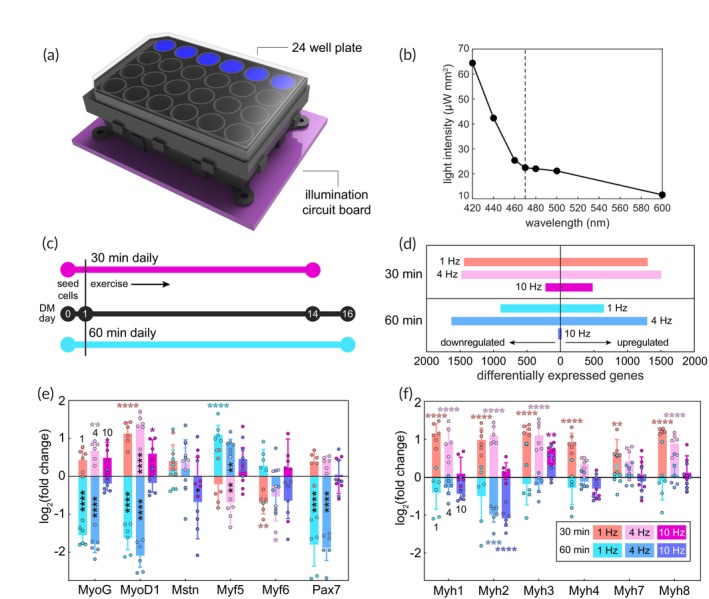
Characterizing transcriptional responses to optical exercise. Including one control condition (six biological replicates) per experiment, each exercise condition comprised six biological replicates, totaling 24 samples for the 30‐min exercise experiment and 24 samples for the 60‐min exercise experiment. (a) High‐throughput optical stimulation platform, adapted from prior work by Repina et al.^29^ (b) Spectral characterization of optical stimulation platform. Dotted line shows intensity at 470 nm, the wavelength typically used to stimulate ChR2(H134R). (c) Tissue culture and experimental timeline for 30‐ and 60‐min exercise experiments. While there is a 2‐day difference in culture timeline for 30‐ and 60‐min exercise studies, qPCR data from previous experiments (Figure S2) show that differentiation dynamics in 2D skeletal muscle monolayers are usually stable after 10 days in culture. (d) Bar graph of significantly upregulated and downregulated gene counts (log_2_(fold change) >0.5, *p*_adj_ <0.05) for each experimental condition. Gene counts obtained by comparing averaged experimental conditions with averaged control conditions. (e) Measured log_2_(fold change) values for genes associated with muscle differentiation and maturation. (f) Measured log_2_(fold change) values for myosin heavy chain (Myh) protein‐encoding genes used to identify muscle fiber types. *p*‐values were calculated using two‐way ANOVA analysis (Prism 10, GraphPad). * Denotes *p*‐value of *p* ≤ 0.05, ** denotes *p* ≤ 0.01, *** denotes *p* ≤ 0.001, and **** denotes *p* ≤ 0.0001. Table S1 for a table of Myh isoforms and their associated fiber types. ANOVA, analysis of variance; qPCR, quantitative polymerase chain reaction.

To conduct high‐throughput studies of muscle optical stimulation, we modified a computer‐controlled optical stimulation platform from Repina et al.^29^ to enable evenly illuminating each well within a standard 24‐well tissue culture plate with 470 nm light (Figure 2a,b) at an intensity of 22.5 μW mm^−2^ (within our established rheobase range). The platform stimulated individual wells with groupings of five LEDs in a pentagonal configuration controlled by a 12‐bit PWM LED Driver (TLC 5947, Adafruit). A single‐board computer (Zero W, Raspberry Pi) sent commands to the LED driver and received programming inputs from a computer via Wi‐Fi connection. Fibrin gels were prepared in the 24‐well plates as described for the rheobase characterization experiment. This compact platform allowed us to exercise tissues inside a standard incubator with tightly controlled temperature, carbon dioxide, and humidity.

In two separate experiments, we exercised tissues daily for 30 min (six biological replicates at 1, 4, and 10 Hz stimulation frequencies, six biological replicates of an unstimulated control) and 60 min (six biological replicates at 1, 4, and 10 Hz stimulation frequencies, six biological replicates of an unstimulated control). These stimulation frequencies and durations were chosen based on previous studies that leveraged similar protocols to enhance contractile force in 3D optogenetic muscle tissues.^17^, ^21^ Stimulation at 1 Hz and 4 Hz represent low‐ and medium‐exertion conditions, respectively, and stimulation at 10 Hz emulates a high‐exertion condition that approaches tetanus but remains within the operating limits of our optical stimulation platform.

Tissues were maintained in 0.6 mL of culture media for the 30‐min exercise experiment and 1.5 mL for the 60‐min exercise experiments, with exercise training commencing within 15 min of fresh media addition. For all tissues, we independently confirmed muscle differentiation efficiency by recording and analyzing contractions under 1 Hz electrical stimulation on day 10, using a function generator (SDG 1032X, Siglent, 10 V, 5 ms pulse width) connected to parallel electrode wires (Figure S10). After ~2 weeks of daily optical exercise stimulation, RNA was extracted and sequenced (Figure 2c).

### 2.3 Bulk RNA‐seq transcript analysis

Bulk RNA‐seq enables analyzing changes in gene expression induced by varying optical exercise training regimens. In both 30‐ and 60‐min exercise experiments, 1 and 4 Hz stimulation yielded comparable numbers of upregulated and downregulated genes (log_2_(FC) >0.5 and *p*_adj_ <0.05), while 10 Hz stimulation produced the lowest number of significantly upregulated or downregulated genes (Figure 2d). In fact, we found that the most significantly downregulated gene in the 10 Hz 60‐min condition was Myh2, the myosin heavy chain protein that identifies type 2a (fast‐oxidative) muscle fibers. Both experiments show clustering of control and 10 Hz‐stimulated tissues, as well as clustering of 1 and 4 Hz stimulated tissues (Figures S3 and S4).

Focusing on protein‐encoding genes associated with muscle fiber maturation and fiber type specification revealed that 1 and 4 Hz stimulation for 30 min significantly upregulated several genes associated with myogenic differentiation, including MyoG and MyoD1, as compared to unstimulated controls (Figure 2e).^41^, ^42^ Moreover, muscles in these exercise conditions upregulated the expression of the four mature myosin isoforms present in mouse muscle, namely Myh1, Myh2, Myh4, Myh7 (Figure 2f). The presence of embryonic (Myh3) and postnatal (Myh8) myosin isoforms indicates that some fibers likely remained in an immature state at the time of RNA extraction. Given the presence of Pax7, a marker of muscle satellite cells with the capacity to differentiate into new muscle fibers, it is possible that new fibers continued to form within exercised tissues at rates comparable to those undergoing fiber type commitment and maturation.

Notably, exercise training for 30 min at 10 Hz stimulation did not have a significant impact on gene expression associated with myogenic differentiation or fiber type commitment. Moreover, exercise training for 60 min yielded significant downregulation of the analyzed muscle‐relevant genes as compared to unstimulated controls.

### 2.4 Gene set enrichment analysis

**FIGURE 3:**
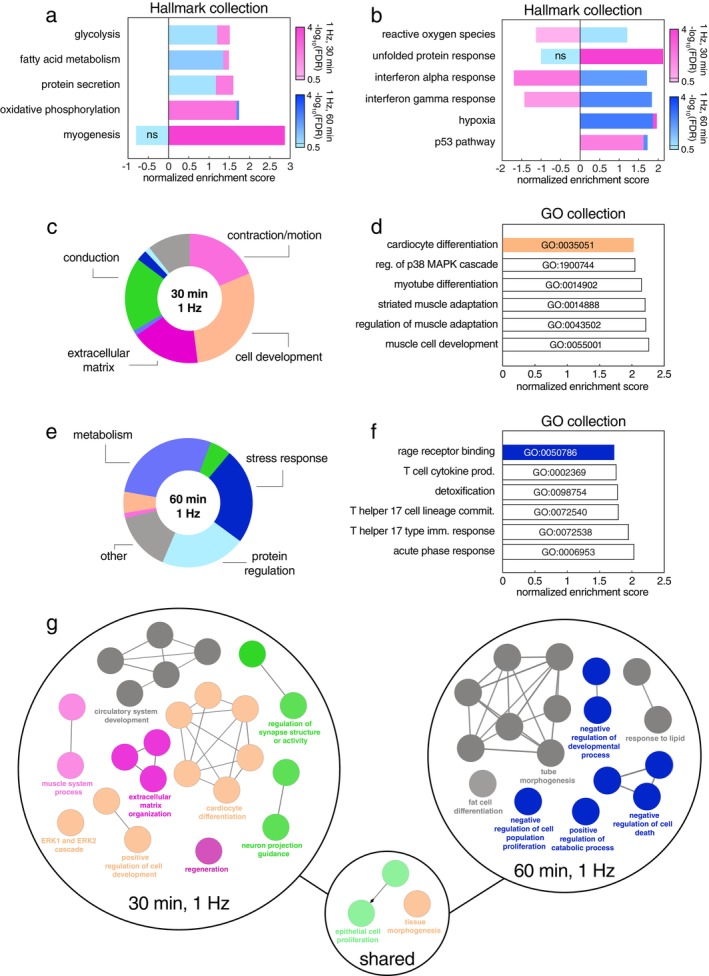
Gene set enrichment analysis (GSEA) results. Normalized enrichment scores obtained by comparing GSEA output of experimental and control groups. Cell transcriptional behavior for 1 Hz conditions from 30‐ and 60‐min experiments are compared using Hallmark and Gene Ontology (GO) gene set collections. Hallmark normalized enrichment scores (NESs) comparing gene sets associated with (a) muscle development and metabolism, and (b) inflammatory responses in both 1 Hz conditions. Lines across the color bars indicate the cutoff for statistical significance, shown on individual bar plots with “ns.” (c) Each of the 75 terms fit into one of eight categories shown in (c) and (e), centered around conduction, contraction, ECM, and cell development processes. (d) Examples of enriched cell development gene sets in the 30‐min condition. (e) Fifty‐five of the 75 most enriched 60‐min GO terms primarily represented metabolic, stress response, and nonspecific protein regulation processes. (f) Examples of terms representing stress response behaviors in the 60‐min condition. (g) CytoGO visualization of gene sets associated with the 250 most upregulated genes from each condition. Grouped terms are color coded as in (c) and (e). Gene sets not associated with a category are colored gray. Shared gene sets are centered between the two experimental groupings.

Looking beyond genes with known roles in muscle maturation, interpretative complications can arise when tying cell function to differential expression of individual genes, especially for regulatory genes implicated in multiple pathways. Gene set enrichment analysis (GSEA) abstracts away from transcriptional granularity and maps aggregate differential expression to all dimensions of cell behavior. Figure 3 explores systemic effects of our exercise training protocols between 1 Hz 30 min and 1 Hz 60 min conditions using Hallmark and Gene Ontology (GO) gene set collections (Biological Process, Molecular Signatures Database, MSigDB). The Hallmark collection comprises 50 well‐defined gene sets that reduce the intrinsic redundancy associated with typical gene set collection reporting^43^ (Figure 3a,b), while GO annotations capture the hierarchical organization of thousands of cellular processes and describe a broad range of cellular activity (Figure 3c–g).^44^ Our GO GSEA focused on biological process terms. Below, we introduce the gene set collections and describe enrichment results, then present more detailed transcriptional trends suggested by both collections.

### 2.5 Hallmark collection GSEA

Figure 3a shows Hallmark snapshots of muscle behavior between 1 Hz 30‐min and 60‐min conditions. Myogenesis, the only muscle‐specific gene set in the Hallmark collection, was the highest‐ranked (most enriched) gene set in the 1 Hz 30‐min condition but was not significantly enriched in the 1 Hz 60‐min condition. Across experiments, myogenesis was significantly enriched in all 30‐min conditions and followed no clear trend in the 60‐min conditions (Figure S5). Gene sets capturing cell metabolic activity included oxidative phosphorylation, glycolysis, and fatty acid metabolism. Clear differences between oxidative and glycolytic metabolism can indicate fiber type transitions, where slow fibers tend to oxidatively synthesize ATP in mitochondria and fast fibers exhibit increased cytosolic glucose consumption.^45^ Oxidative phosphorylation ranked in the top five gene sets for all tested conditions except the 1 Hz 30‐min and 10 Hz 60‐min conditions.

The Hallmark collection also tracks immune and stress response‐related transcriptional activity, and Figure 3b shows gene sets relevant to muscle development and function. Except for reactive oxygen species (ROS), both 1 Hz 30‐ and 60‐min conditions have comparable normalized enrichment scores (NESs) for the presented gene sets. Overall, the 1 Hz 30‐min condition exhibited lower enrichment of immune‐related gene sets than the 1 Hz 60‐min condition. For example, interferon alpha and gamma responses were significantly de‐enriched in the 1 Hz 30‐min condition and significantly enriched in the 1 Hz 60‐min condition. Exercise‐induced stresses can complicate distinctions between myogenic and pathological responses, especially in differentiating muscle tissues. The GO collection provides additional granularity in the form of over 15,000 gene sets to help clarify these distinctions.

### 2.6 GO collection GSEA

To explore a wider range of cellular processes, we investigated the 75 highest‐enriched GO gene sets for each experimental condition and categorized relevant terms into one of the eight categories seen in Figure 3c,e. Figure S6 shows plots of gene sets from the other categories. The 1 Hz 30‐min condition had the greatest frequency of contraction, developmental, conduction, and extracellular matrix (ECM) gene sets. Conduction‐related gene sets primarily indicate protein expression related to transmembrane ion transport, but we also included neuron morphology gene sets such as dendrite morphogenesis (GO: 0048813) due to their low incidence in our data. ECM gene sets indicate general matrix reorganization and collagen synthesis processes, including one basement membrane organization term (GO: 0071711) that is more relevant to muscle fiber adhesion. Of the terms we explored, ECM terms were unique to the 1 Hz 30‐min condition. Interestingly, we observed that during RNA extraction, tissues in the 1 Hz 30‐min condition were the most difficult to scrape away from the underlying fibrin hydrogel, suggesting that upregulation of ECM remodeling was associated with stronger cell‐matrix adhesion. These observations align with previous studies of C2C12 cells demonstrating that 1 Hz electrical stimulation increases collagen expression relative to unstimulated controls.^31^ Figure 3d shows examples of enriched cell development gene sets, mostly pertaining to muscle differentiation and adaptation. Multiple cardiac contraction, morphogenesis, and development terms are present in GSEA results, as expected given the set of shared contractile and conductive proteins between skeletal and cardiac muscle tissues.^46^

In contrast to the 1 Hz 30‐min condition, the 1 Hz 60‐min GO annotations presented a large proportion of enriched stress‐response, metabolic, and protein regulation gene sets (Figure 3e,f). Metabolic gene sets included several subprocesses associated with mitochondrial complex formation and oxidative metabolism. Protein regulation gene sets captured ribosome assembly and protein membrane targeting. Similar gene set groupings given for other exercise conditions are presented in Figure S7.

To confirm our manual gene set groupings and investigate relationships between the “other” gene sets, we used ClueGO, a Cytoscape plugin, as an alternative to obtain nonredundant GO classifications (Figure 3g).^47^ This visualization technique identified tube morphogenesis and circulatory system development relationships, possibly relating to angiogenic, neural, and nephric terms identified in Hallmark and GO analysis. The 1 Hz 60‐min condition also included lipid, liver, and adipose‐associated classifications, possibly implicated in metabolic signaling pathways and membrane modifications.

### 2.7 Hypoxia

Contracting muscle has oxidative metabolism 10–100 times higher than resting muscle,^48^ so it is reasonable that even a monolayer of skeletal muscle fibers can outpace passive oxygen diffusion through a column of media several millimeters in height. Given the occurrence of spontaneous twitch in our monolayers, baseline oxygen consumption is likely higher than expected for resting muscle tissue. A previous study has shown that incubation under 5% O_2_ caused muscle atrophy, and more intermediate 10%–15% O_2_ incubation supported differentiation and hypertrophy in C2C12 tissue constructs.^49^ Yet, Figure 3b reveals differences in tissue response to hypoxic and exercise‐based stressors. The 1 Hz 30‐min condition had significantly downregulated ROS and interferon responses (implicated in muscle injury and repair), while the 1 Hz 60‐min condition was upregulated in these pathways.

### 2.8 Channelrhodopsin expression and phototoxicity

To investigate the effect of optical stimulation on ChR2(H134R) expression in our tissues, raw read counts were obtained by mapping sequencer output to a modified reference genome containing previously described ChR2(H134R)‐tdTomato‐WPRE plasmid maps.^50^ Interestingly, the 1 Hz 30‐min condition demonstrated significantly higher ChR2(H134R) expression as compared to unstimulated controls, in line with the trends seen in conduction gene set enrichment (Figures S7, S9).

Importantly, while long‐wavelength (red and infrared) light has previously been shown to have positive photobiomodulatory effects on muscle tissue in vitro,^51^ short‐wavelength light (the range typically used for optogenetic studies) can damage cells by causing ROS to form within the culture media and cells.^52^ Yet, exercising muscle tissues also generate ROS and can complicate the relative contributions of phototoxicity and exercise to downstream transcriptional data.^53^ The Hallmark “reactive oxygen species pathway” gene set was among the lower‐ranked enriched gene sets, following no clear trend across conditions. Among the GO terms we considered in this study, only the 10 Hz 60‐min condition contained a gene set specific to ROS.

The Hallmark collection also includes two gene sets devoted to upregulation and downregulation of genes related to UV exposure. UV light includes wavelengths up to 400 nm, nearing the 470 nm wavelength used to activate ChR2(H134R). Figure S8 shows a plot of “UV response up” and “UV response dn” (down) gene set enrichment over all conditions, showing no obvious trends between light exposure and gene set upregulation or downregulation in our studies.

## 3 DISCUSSION

Optical stimulation of skeletal muscle, when coupled with high‐throughput culture and stimulation formats, offers a minimally invasive and scalable method to study tissue response to exercise. While a few prior studies show preliminary evidence that stimulating optogenetic muscle tissues with light can enhance force production, no studies, to our knowledge, have reported rheobase for optogenetic muscle or characterized how various exercise regimens broadly modulate muscle gene expression. In this study, we leverage a 2D muscle monolayer platform and open‐source optical stimulation hardware to evenly illuminate all cells within an engineered tissue. Our rigorous characterization experiments show that rheobase in widely used optogenetic C2C12 lines^17^, ^21^, ^23^, ^26^, ^34^, ^35^ can be achieved at light intensities between 5 and 25 μW mm^−2^, one to two orders of magnitude lower than intensities used in prior studies. We attribute this finding to the fact that prior optogenetic muscle stimulation studies likely either used parameters that were based on in vivo studies or used light sources without adjustable intensities.

### 3.1 Spontaneous twitch

Several in vitro studies of skeletal muscle have reported that differentiated tissues routinely spontaneously initiate contractions even in the absence of motor neuron innervation.^54^, ^55^, ^56^, ^57^ Spontaneous contraction is a necessary step in myogenesis, as intermittent twitches help align contractile and structural proteins during sarcomere synthesis.^58^ Furthermore, spontaneous contractions provide the necessary signals for embryonic neuromuscular junction formation.^59^, ^60^ Even adult muscle, when denervated, has the potential to develop spontaneous contractions.^61^

In our experiments, we observed continuous spontaneous twitch in muscle tissues across all conditions (even in the absence of ambient light exposure). This observation implies that our tissues may effectively exercise themselves even in the absence of optical stimulation. Thus, while our control conditions are unstimulated, they are not necessarily unexercised. Future studies that modulate spontaneous twitch frequency by delivering low intensity, sustained light exposure (see Figure 1g) throughout the entire study could account for this factor by precisely controlling spontaneous twitch across all tested conditions.

### 3.2 Transcriptomic analysis

Despite the presence of spontaneous twitch in all conditions, our optical exercise protocols still produce meaningful differential gene expression results. RNA‐seq data reveal similar trends in cell behavior when comparing individual genes (Figure 2e,f) and GSEA results (Figure 3). Overall, transcriptomic analysis points to myogenic responses in the 1 Hz 30 min and 4 Hz 30 min experiments and pathological stress responses in the 60‐min experiment across all tested stimulation frequencies.

Discussing “stress response” in exercise contexts is difficult because exercise itself is a form of stress. Hypoxia is another form of stress, and GSEA results show significant enrichment of hypoxia in both experiments (Figure S5). Yet, hypoxia has a multidimensional role in exercise contexts, being itself an expected consequence of exercise. At the right dose, hypoxia supports myogenesis, muscle hypertrophy, and fiber type transitions,^49^, ^62^ motivating future studies that carefully measure and control hypoxia in muscle exercise studies.

We observed clustering between 1 and 4 Hz 30‐min conditions, which is reasonable given that both frequencies stimulate independent twitches in muscle.^17^, ^63^, ^64^ By contrast, muscle fibers undergoing 10 Hz stimulation (near‐tetanic contraction) do not undergo full cycles of contraction and relaxation, possibly incurring less wear on fibers due to smaller displacements. This could serve as one possible explanation for transcriptional similarities between the 10 Hz 30‐min conditions and unstimulated controls. Alternatively, it is possible that muscles undergoing 10 Hz stimulation fatigued more quickly, and thus did not actively contract for a significant portion of the exercise training regimen.

### 3.3 Technical limitations and future directions

Our study, similar to most other in vitro tissue culture studies, relied on relatively static culture media conditions (i.e., media replenished once a day) and passive oxygen diffusion. Given that exercise in vivo occurs in the presence of active nutrient and oxygen perfusion, future high‐throughput studies that measure and control for oxygen and glucose consumption may enable more physiologically relevant models of exercise and perhaps highlight benefits of training regimens longer than 30 min. Fortunately, given that optical stimulation platforms (such as the one described in this study) are compact and can perform non‐contact stimulation of cells through plastic‐ and glass‐bottom well plates, they are compatible with integrating other equipment that enables active media perfusion and oxygen generation.

## 4 CONCLUSIONS

We have combined techniques for longitudinal culture of 2D muscle monolayers with a high‐throughput optical stimulation apparatus to build a scalable and minimally invasive exercise assay platform. Our methodology enabled characterizing the minimal light intensity required to activate optogenetic skeletal muscle cells, and mapping diverse changes in muscle gene expression in response to exercise training at various frequencies (1–10 Hz) and durations (0–60 min). Future studies that integrate automated culture systems for glucose and oxygen delivery may further enhance the physiological relevance of our platform. Likewise, studies that integrate co‐cultures of muscle with other cell types may lend insight into the systemic biochemical and mechanical impacts of exercise on other tissues. We anticipate that insights from this study will inform optimized exercise protocols for promoting maturation of tissue engineered skeletal muscle, with potential applications in disease modeling, regenerative medicine, and biohybrid robotics.^65^, ^66^, ^67^, ^68^

## 5 MATERIALS AND METHODS

### 5.1 Culture substrate preparation

Culture substrates were prepared similar to prior work.^28^ For the optical characterization experiment, six‐well plates were fitted with 15 mm‐diameter acrylic wells. Fibrin hydrogel prepolymer was prepared by first mixing a solution of DMEM (Sigma Aldrich, D6429) with 8 mg/mL fibrinogen from fetal bovine plasma (Sigma Aldrich, F8630‐1G). Then, 4 μL/mL thrombin from bovine plasma (Sigma‐Aldrich, T4648‐1KU) was added to the solution, mixed, and pipetted into the acrylic wells at 850 μL per well. As the fibrin gel cured, stamps with 25 μm groove widths were aligned with the acrylic well and placed at the top of the gels to replicate the groove pattern on the gel surface.

Stamps were prepared using prior fabrication methods previously described.^28^ Prior to gel insertion, stamps were sterilized overnight in 70% ethanol and with UV radiation the day of fibrin preparation. To prevent adhesion to fibrin, the stamps were first soaked for 15 min in bovine serum albumin (BSA, Thermo Fisher Scientific, 37525) diluted to a 5% vol/vol concentration. Immediately before gel insertion, stamps were removed from BSA and lightly dried with an aspirator to minimize the BSA layer. Then, a small amount of phosphate‐buffered saline (Thermo Fisher Scientific, 20012027) was placed on the stamp surface to coat the grooves and minimize trapped air when submerged in the fibrin prepolymer. Gels cured for 1 h at room temperature and were maintained in DMEM after stamp removal. Stamps were then cleaned with soap and water using a thin‐bristle toothbrush to de‐soil the stamp face and sides, then underwent sonication in 70% ethanol for 30 min. For the exercise experiments, 24‐well plates were prepared identically to the 6‐well plates except that no acrylic well was used and each well had 500 μL of fibrin.

### 5.2 Cell culture

Optogenetically engineered C2C12 mouse myoblasts (expressing 470 nm blue‐light sensitive ChR2(H134R) tagged with tdTomato) were used for all experiments.^17^ We followed standard tissue culture protocols; ethical considerations were not required as we used an established mouse cell line and did not use cells from any human donors in this study. Cells were expanded in growth medium (GM) consisting of fetal bovine serum (1:10 vol/vol, Life Technologies, A5670701), Corning™ L‐glutamine solution (1:100 vol/vol, Fisher Scientific Co LLC, MT25005CI), and 1% penicillin–streptomycin (Fisher Scientific Co LLC, MT30002CI) dissolved in DMEM. The cells were seeded on stamped gels at a density of 100,000 cells per well (for both 6 and 24‐well plates) and grown in GM with 6‐aminocaproic acid (ACA) (Sigma‐Aldrich, A2504‐100G) at a ratio of 1:50 vol/vol for 1 day until they reached confluency. They were then switched to differentiation medium (DM) consisting of horse serum (1:10 vol/vol, Gibco, 26050‐088), Corning™ L‐glutamine solution (1:100 vol/vol, Fisher Scientific Co LLC, MT25005CI), and 1% penicillin–streptomycin (Fisher Scientific Co LLC, MT30002CI) in DMEM (Sigma Aldrich, D6429), supplemented with insulin‐like growth factor 1 (1:20,000 vol/vol, PeproTech, 100‐11R3‐1MG) and ACA. Cells in 6‐well plates were maintained in 4 mL of media and cells in 24‐well plates were maintained in 0.6 mL of media (30 min exercise) and 1.5 mL of media (60 min exercise).

### 5.3 Optical characterization experiment

Cells were seeded in a six‐well plate and cultured for 9 days in DM without exercise before undergoing characterization. On DM day 9, optically stimulated cell contractions were filmed using an Axiocam 202 mono camera (Zeiss) connected to an inverted brightfield microscope (Primovert, Zeiss). To produce the optical pulses, a function generator (SDG 1032X, Siglent) modulated input signals from an LED driver (LEDD1B, Thorlabs) to a collimated fiber‐optic LED system (M470F3, Thorlabs), previously described.^64^ The collimating lens (F950FC‐A, Thorlabs) was inserted sideways into a fused deposition modeling 3D printed fixture that reflected light downwards to the tissue to avoid collisions with the microscope's overhead lamp and maintain spotlight perpendicularity. The fixture included alignment elements which centered the light over each well. Optical intensity was measured with a photometer (PM100D, S130C, Thorlabs) calibrated to the same distance from the light as tissues in the well plate. Plates were illuminated with a 350‐lumen red LED hunting flashlight (X.YShine) to avoid unintended stimulation. To gather data, videos were taken at 30 fps of contractile responses to single light pulses while sequentially increasing the intensity, repeating for each well. Displacement fields and mean average displacement approximations of tissue contraction were generated from video frames using an open‐source tracking algorithm, described previously.^37^, ^69^ A custom MATLAB script, specifically utilizing the “findpeaks” function, determined maximum displacement amplitudes for twitch and stimulated contractions used in Figure 1. One caveat of this analysis is that the optical pulses briefly saturate the camera, rendering the tracking software unable to track illuminated frames. Since we are interested in what happens after illumination, we track frames backwards in time up to the last frame of illumination to ensure tracking continuity.

### 5.4 Exercise study

All conditions were exercised daily, starting on DM day 1. Two open‐source optical illumination plates were constructed and kept inside an incubator to facilitate high‐throughput stimulation.^29^, ^30^ The light guide attachments were adjusted in CAD (SolidWorks 2021) to fit the dimensions of our well plate (Cellvis: #P24‐1.5H‐N). Experiments were conducted in 24‐well plates with sxi biological replicates per condition (control, 1, 4, 10 Hz). The illumination platform saves stimulation settings on an integrated Raspberry Pi Zero W computer, so settings were loaded once through the included “JarJarBlinks” Java applet GUI. Media was changed daily, no more than 15 min before exercise.

### 5.5 Quantitative polymerase chain reaction

Reverse transcription of RNA into cDNA was conducted with the High‐Capacity cDNA Reverse Transcription Kit (Applied Biosystems). Technical replicates were prepared for quantitative polymerase chain reaction (qPCR) with TaqMan Fast Advanced Master Mix and TaqMan Assays for the genes of interest (Applied Biosystems), and reaction amplification curves were obtained using a CFX Opus 96 (Bio‐Rad). Relative gene expression was quantified with the ΔΔCt method, using GAPDH as a reference gene.^70^

### 5.6 RNA sequencing

RNA extraction was performed on day 14 of muscle differentiation for the 30‐min experiment and day 16 for the 60‐min experiment using the Qiagen RNeasy Mini Kit (Hilden, Germany) following the manufacturer's instructions. Cell monolayers were treated with the lysis buffer for 15 min before being spun down in QIAshredder spin columns to homogenize the tissue.

Sequencing libraries were generated using the NEBNext® Ultra™ II Directional RNA preparation with poly(A) selection kit. Library sizes were quantified and verified via qPCR and Fragment Analyzer before being loaded on the Singular G4 platform in a 50‐base paired‐end configuration with 8 + 8 nucleotide indexes.

Reads were mapped against mm39 (GRCMm39) using the BWA‐MEM algorithm.^71^ Quality control metrics such as mapping rates, unique 20‐mers, and fraction of ribosomal RNAs were calculated using bedtools version 2.30.0. FASTQ files were processed using the nf‐core/rnaseq version 3.11.1 pipeline. We utilized the GRCm39 reference genome and ENSEMBL GRCm39 murine annotations.

Differential gene expression was done in DESeq2 in R version 4.2.0 using the apeglm method to provide Bayesian shrinkage estimators for effect sizes.^72^ We reported log_2_ fold changes and Benjamini‐Hochberg adjusted *p*‐values. Our GSEA was done in version 3.0. We utilized the log_2_ fold changes and *p*‐values from DESeq2 to rank the genes in preparation for analysis in the GSEAP reranked module. Two major MSigDB collections were used to investigate transcriptional differences, Hallmark and C5 (GO) gene set collections. We reported NESs from the GSEA analysis with their false discovery rate (FDR) *q*‐values. Gene sets with FDR *q*‐values <0.25 were considered statistically significant.

### 5.7 Statistical analysis

Statistical methods used to evaluate significance are defined in accompanying figure captions.

## AUTHOR CONTRIBUTIONS

Conceptualization: Ronald Heisser, Angel Bu, Tamara Rossy, Ritu Raman. Methodology: Ronald Heisser, Laura Schwendeman, Tamara Rossy, Ritu Raman. Investigation: Ronald Heisser, Angel Bu, Laura Schwendeman, Tamara Rossy, Pavankumar Umashankar, Vincent Butty, Ritu Raman. Formal analysis: Ronald Heisser, Angel Bu, Pavankumar Umashankar, Vincent Butty, Ritu Raman. Visualization: Ronald Heisser, Angel Bu. Data curation: Vincent Butty. Funding acquisition: Ritu Raman. Project administration: Ritu Raman. Supervision: Ritu Raman. Writing – original draft: Ronald Heisser, Angel Bu, Ritu Raman. Writing – review and editing: Ronald Heisser, Ritu Raman.

## CONFLICT OF INTEREST STATEMENT

The authors declare no conflicts of interest.

## Supporting information


**Data S1:** Supplementary information.

**Video S1:** Twitch response from Figure 1d.

**Video S2:** Frequency modulation with constant illumination.

## Data Availability

The data discussed in this publication have been deposited in NCBI's Gene Expression Omnibus^73^ and are accessible through GEO Series accession number GSE306860.
