# Supplementary material for: Physiological and functional characterization for high‐throughput optogenetic skeletal muscle exercise assays
Source: Bioeng Transl Med. 2025 Dec 14;11(3):e70101. doi: 10.1002/btm2.70101 (PMC13247415; doi:10.1002/btm2.70101)
Supplement: Supplementary file 1 — Data S1: Supplementary information. [file BTM2-11-e70101-s003.docx]

**Supplementary Information**


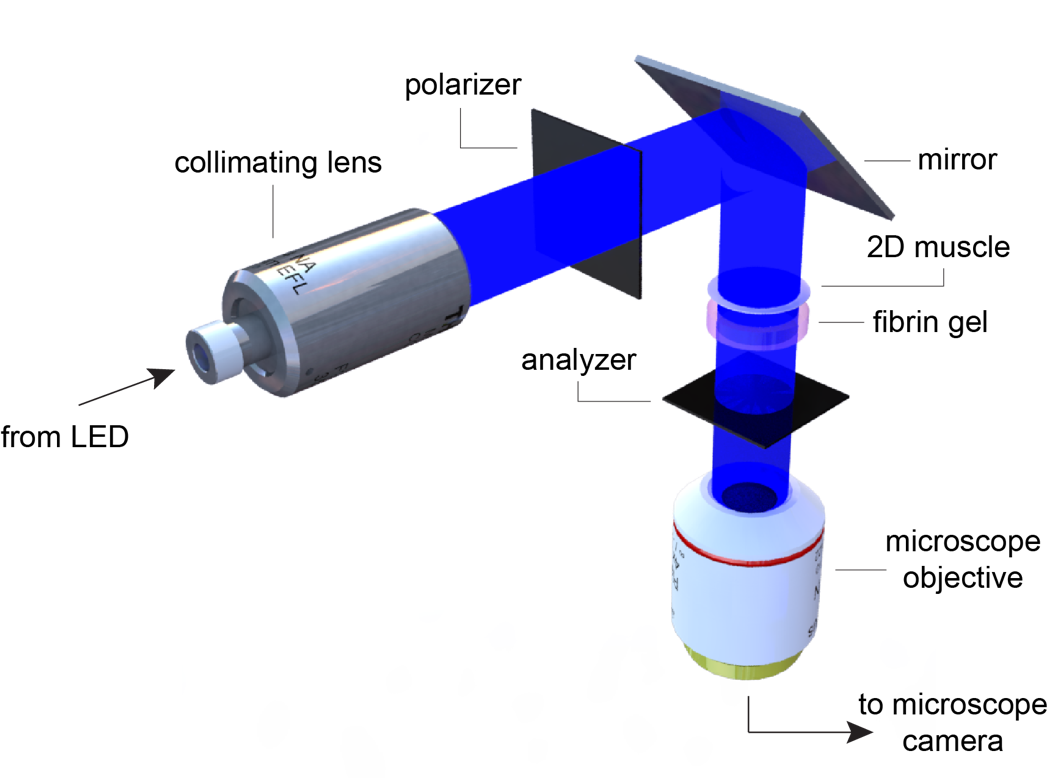


Figure S1: Optical stimulation setup used to characterize optogenetic muscle rheobase.


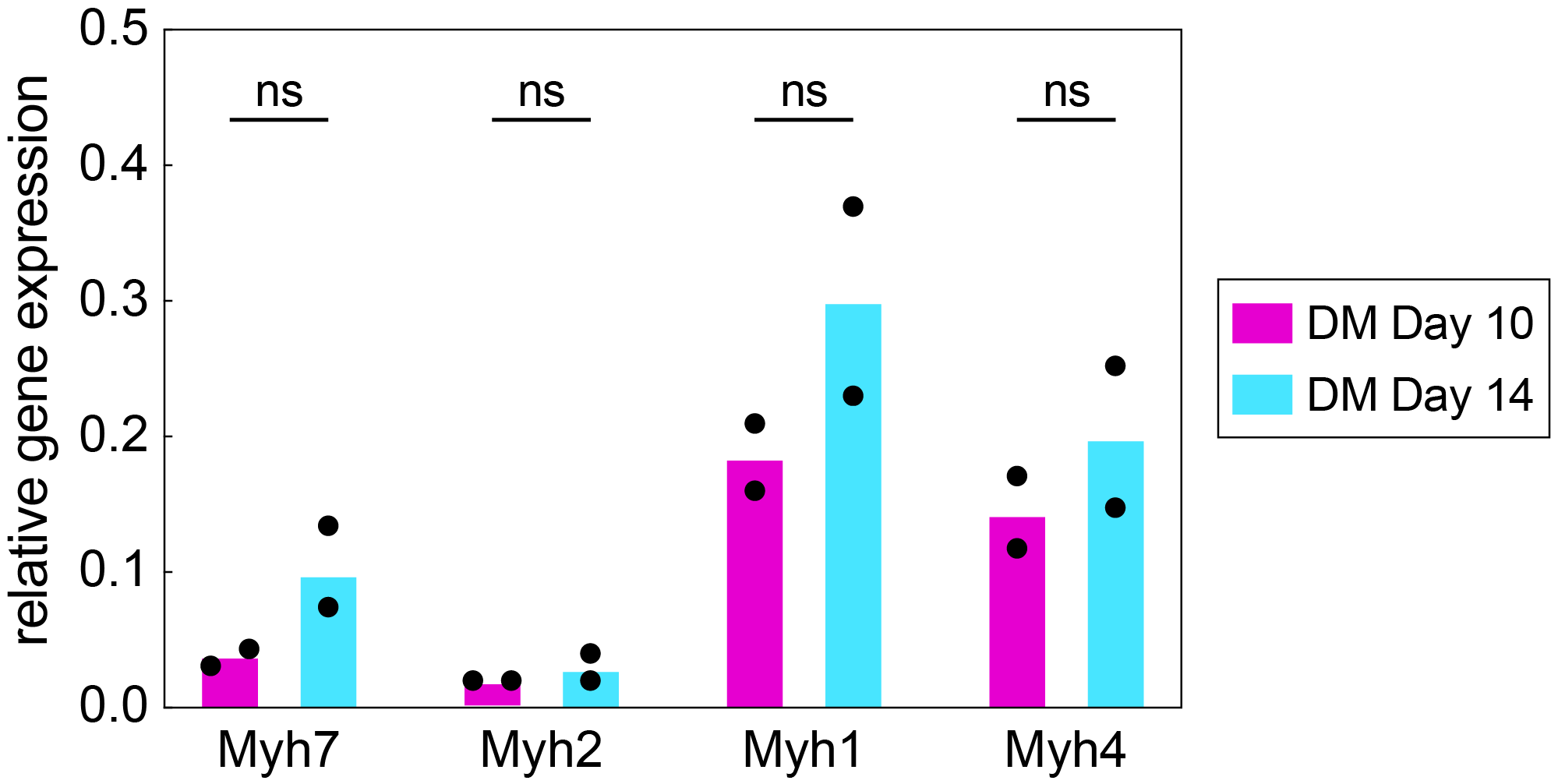


Figure S2: qPCR comparison of gene expression showing stable expression of mature myosin isoforms after 10 days in differentiation media. Relative gene expression values were obtained by calculating 2^-ΔΔCt^ of three technical replicates for two biological replicates. Averages of target genes were compared against averages of a housekeeping gene (MYOG) for each experiment.


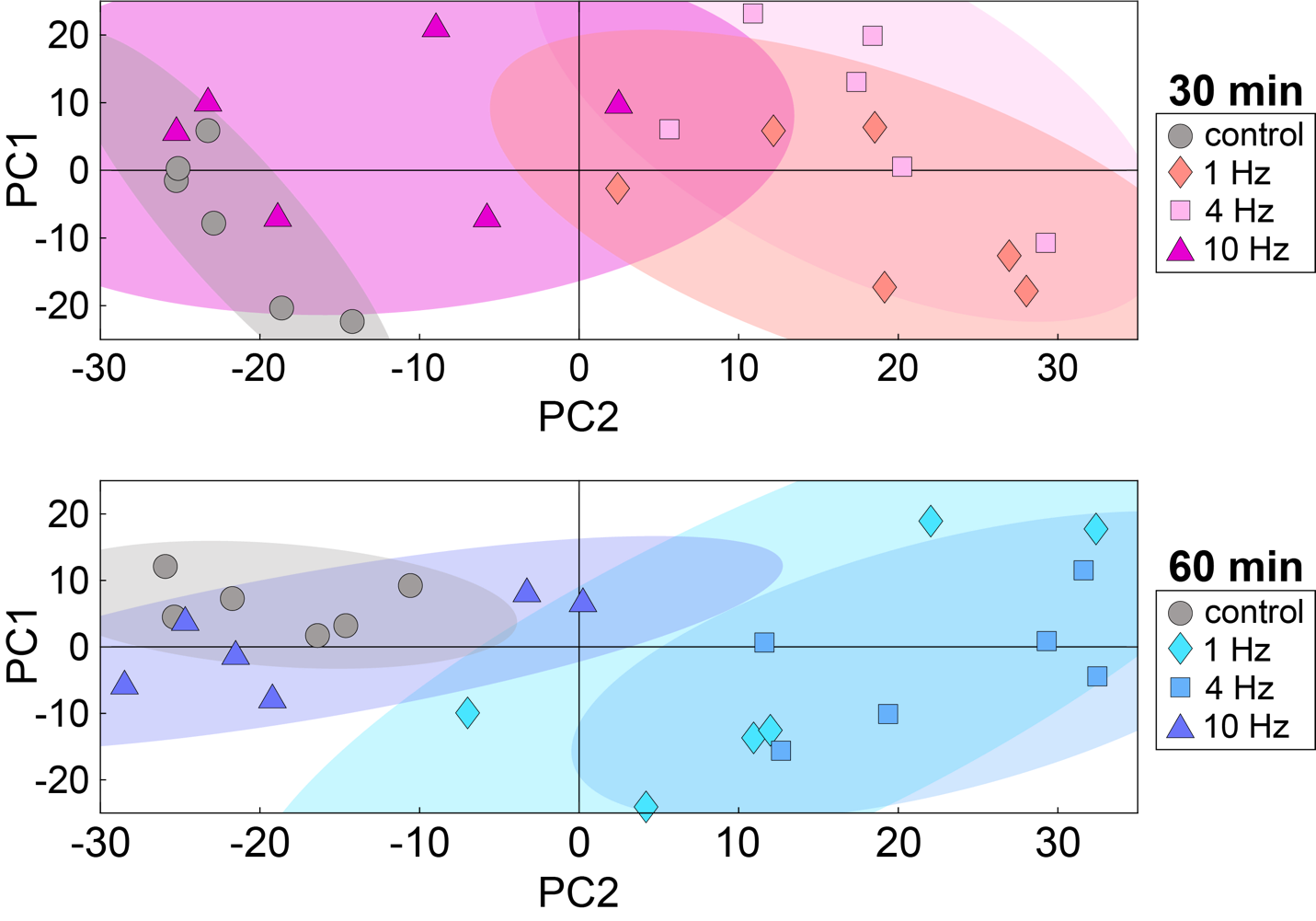


Figure S3: Principal component analysis (PCA) plots for 30 minute and 60 minute exercise experiments. Error ellipses (95% confidence interval) were plotted using “error_function” in MATLAB^1^.


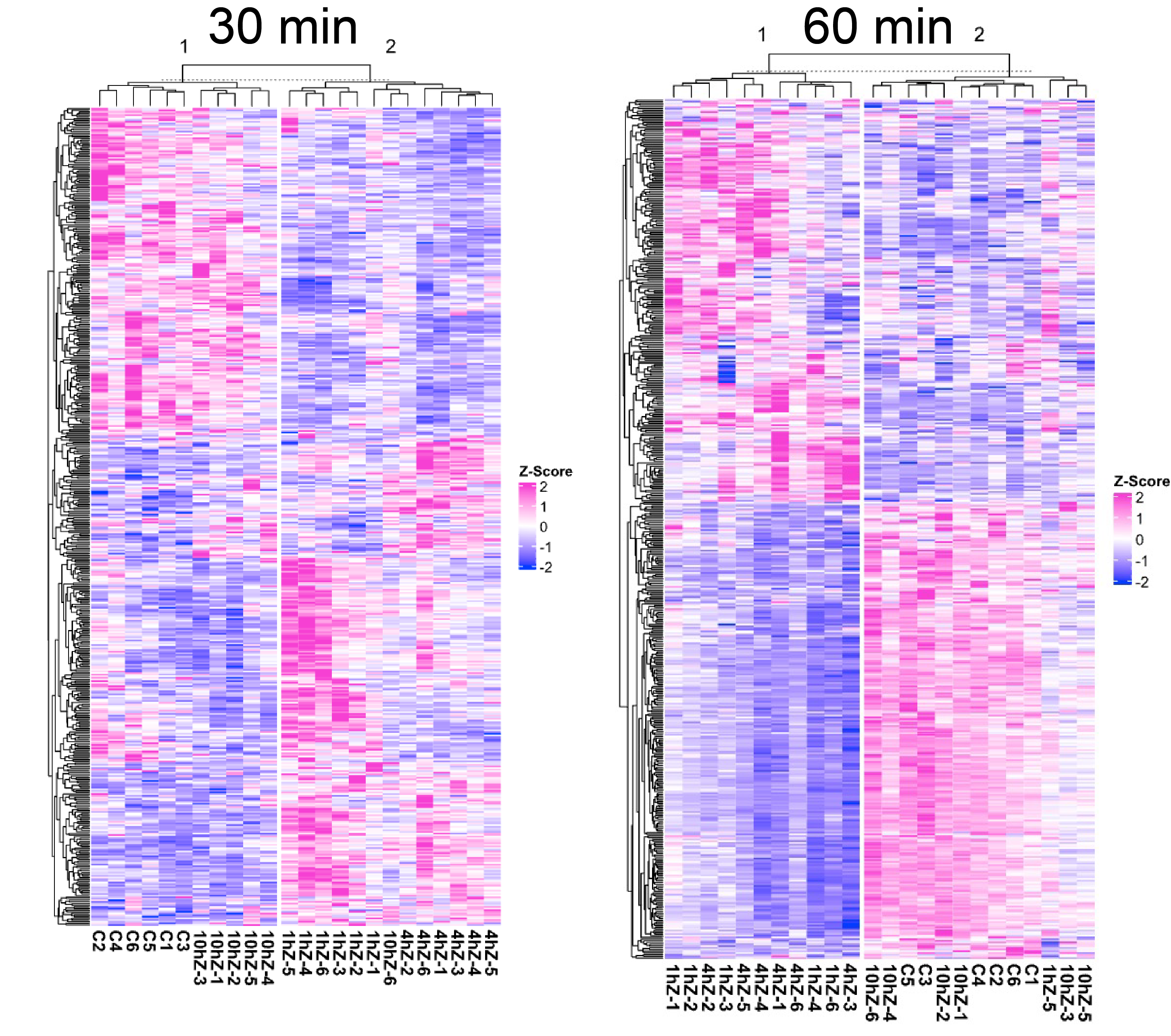


Figure S4: RNA-seq heatmaps. Heatmaps show top 500 differentially expressed genes. Genes were hierarchically clustered on a z-score normalized matrix of transcripts per million. “C” indicates control conditions.


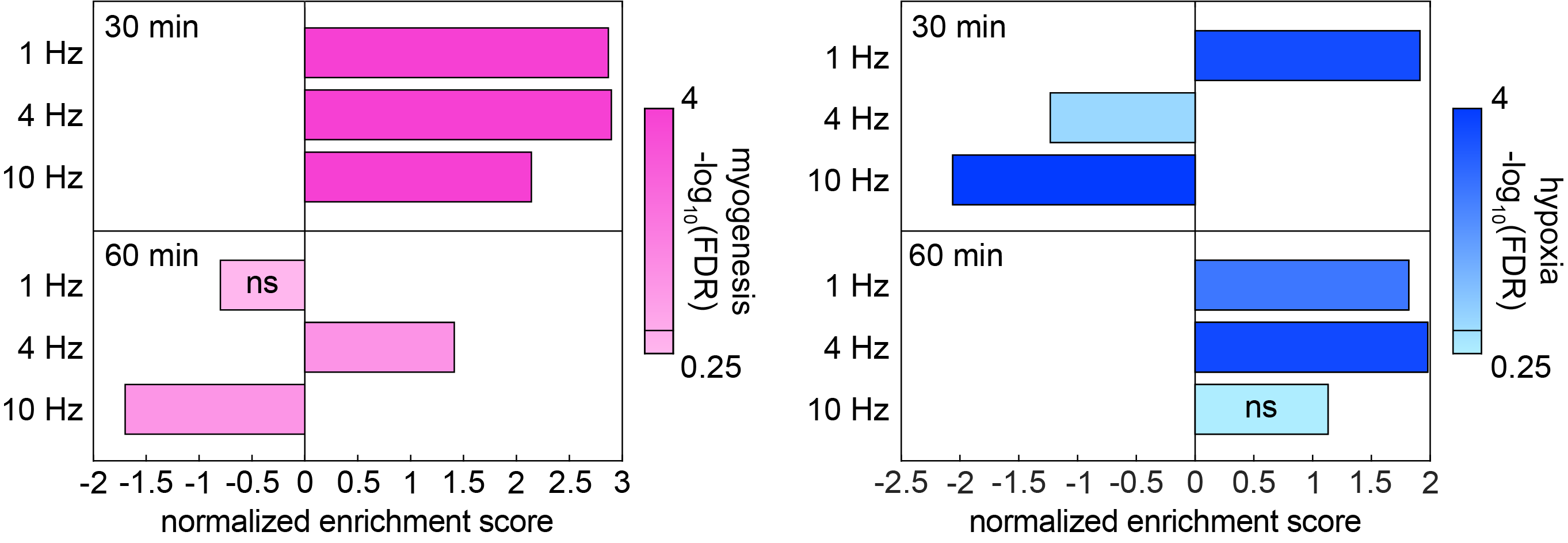


Figure S5: Plots of Hallmark “myogenesis” (left) and “hypoxia” (right) gene sets over all experimental conditions. Normalized enrichment score obtained by comparing GSEA output of grouped experimental and control conditions. Conditions marked with “ns” have -log_10_(FDR) < 0.25, thus are considered not statistically significant.


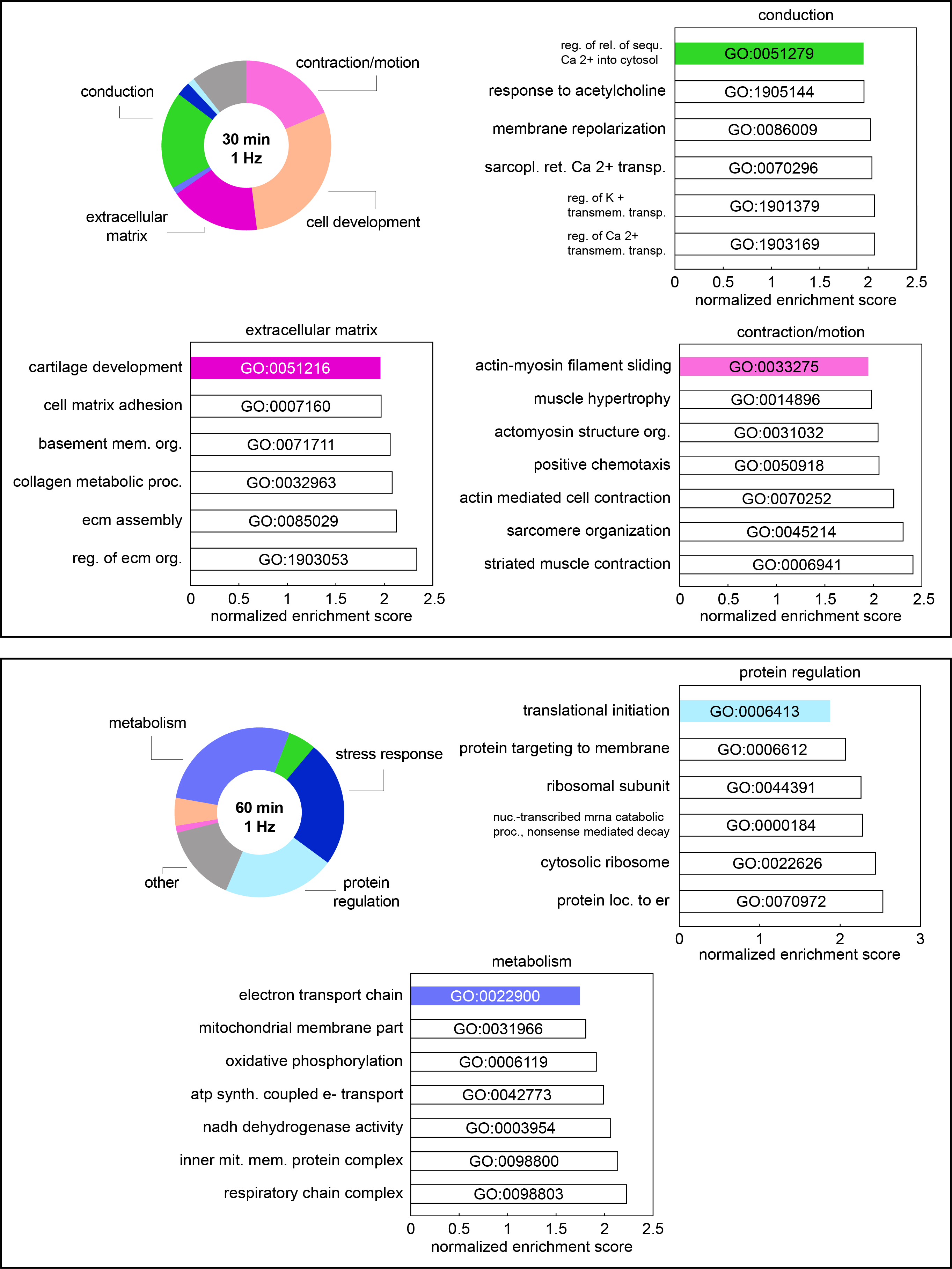


Figure S6: Extended 1 Hz GO term plots from Figure 3.


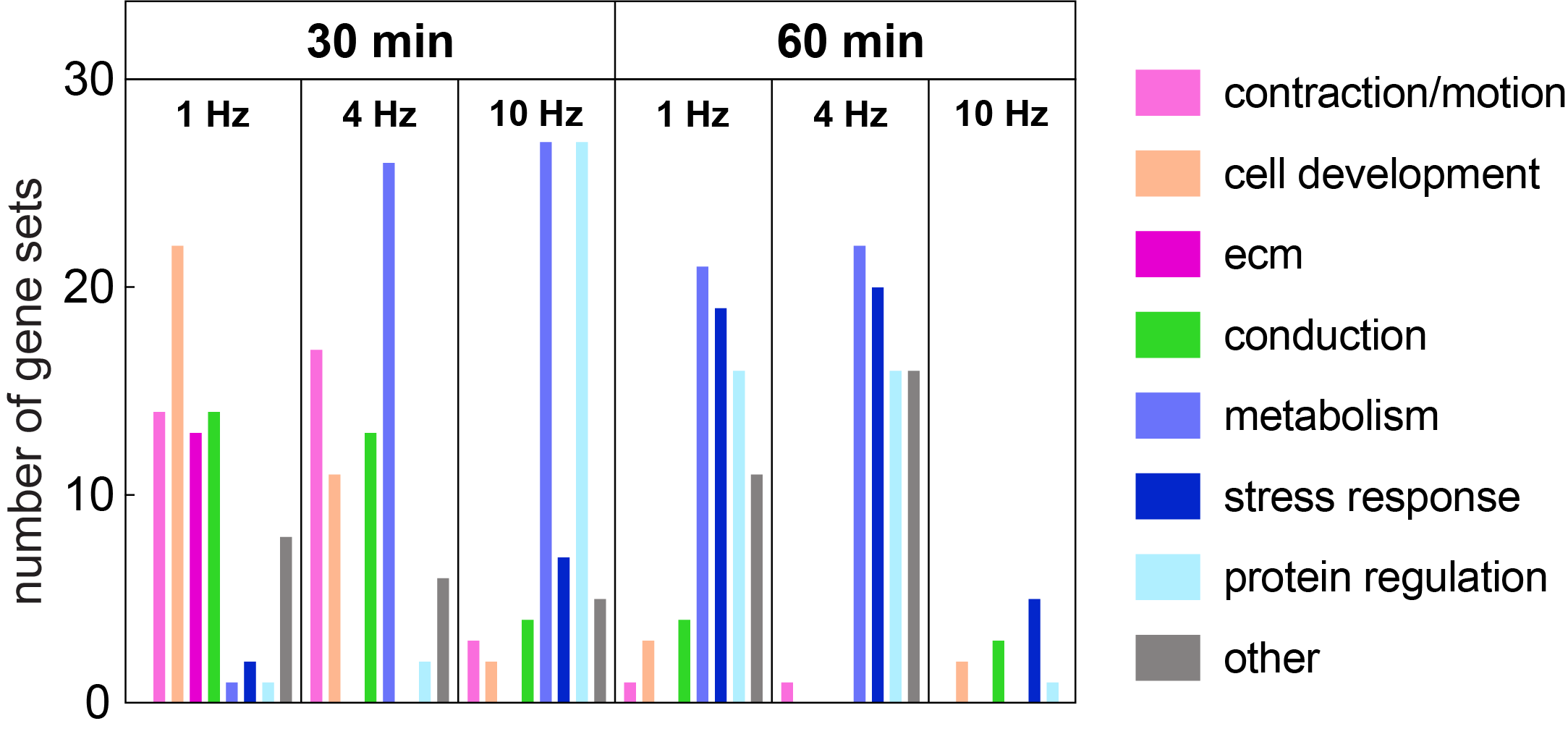


Figure S7: GO term frequency comparison across experiments.


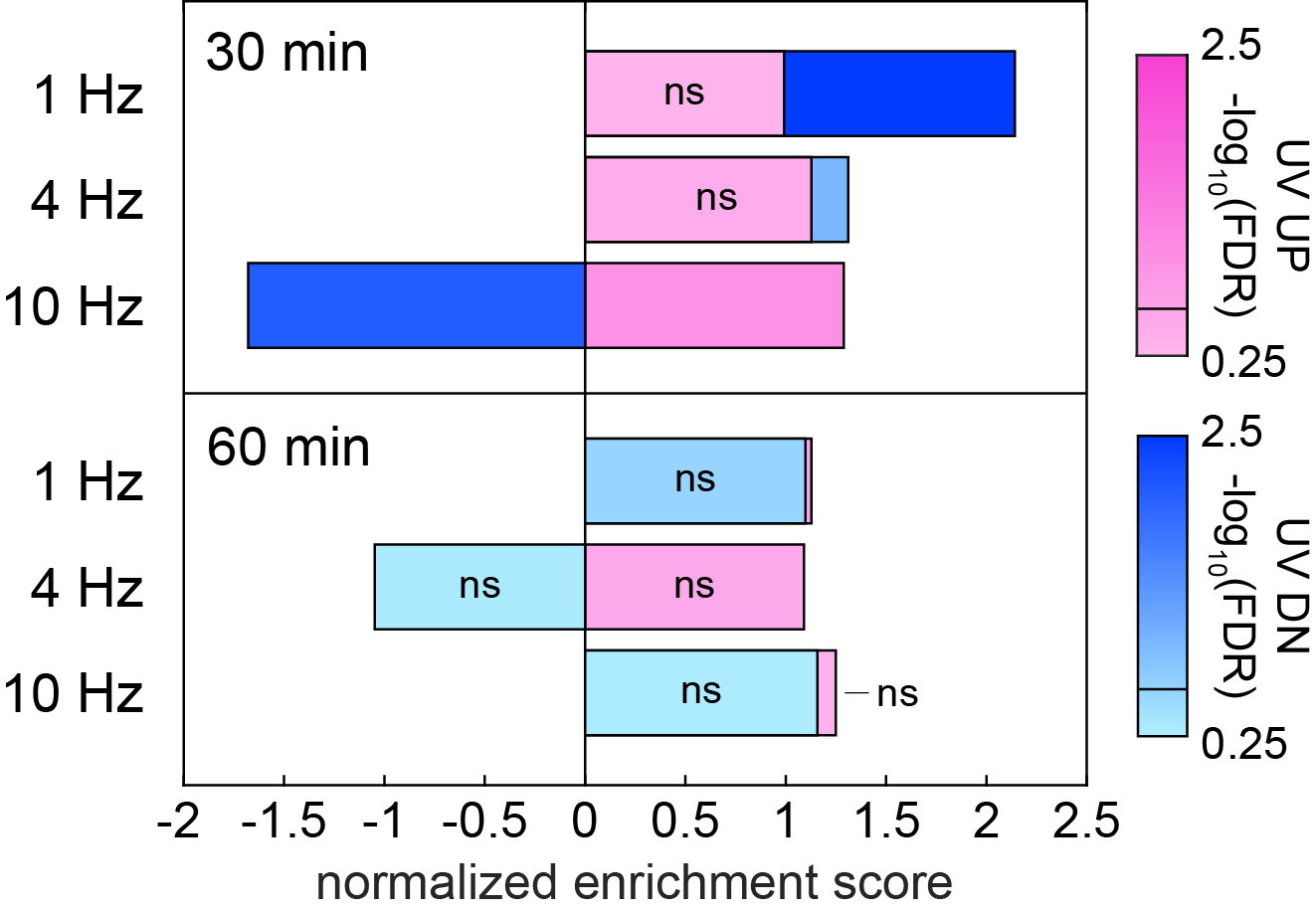


Figure S8: UV response up (UV UP) and UV response down (UV DN) Hallmark gene set cross-comparison. Normalized enrichment score obtained by comparing GSEA output of grouped experimental and control conditions. Conditions marked with “ns” have -log_10_(FDR) < 0.25, thus are considered not statistically significant.


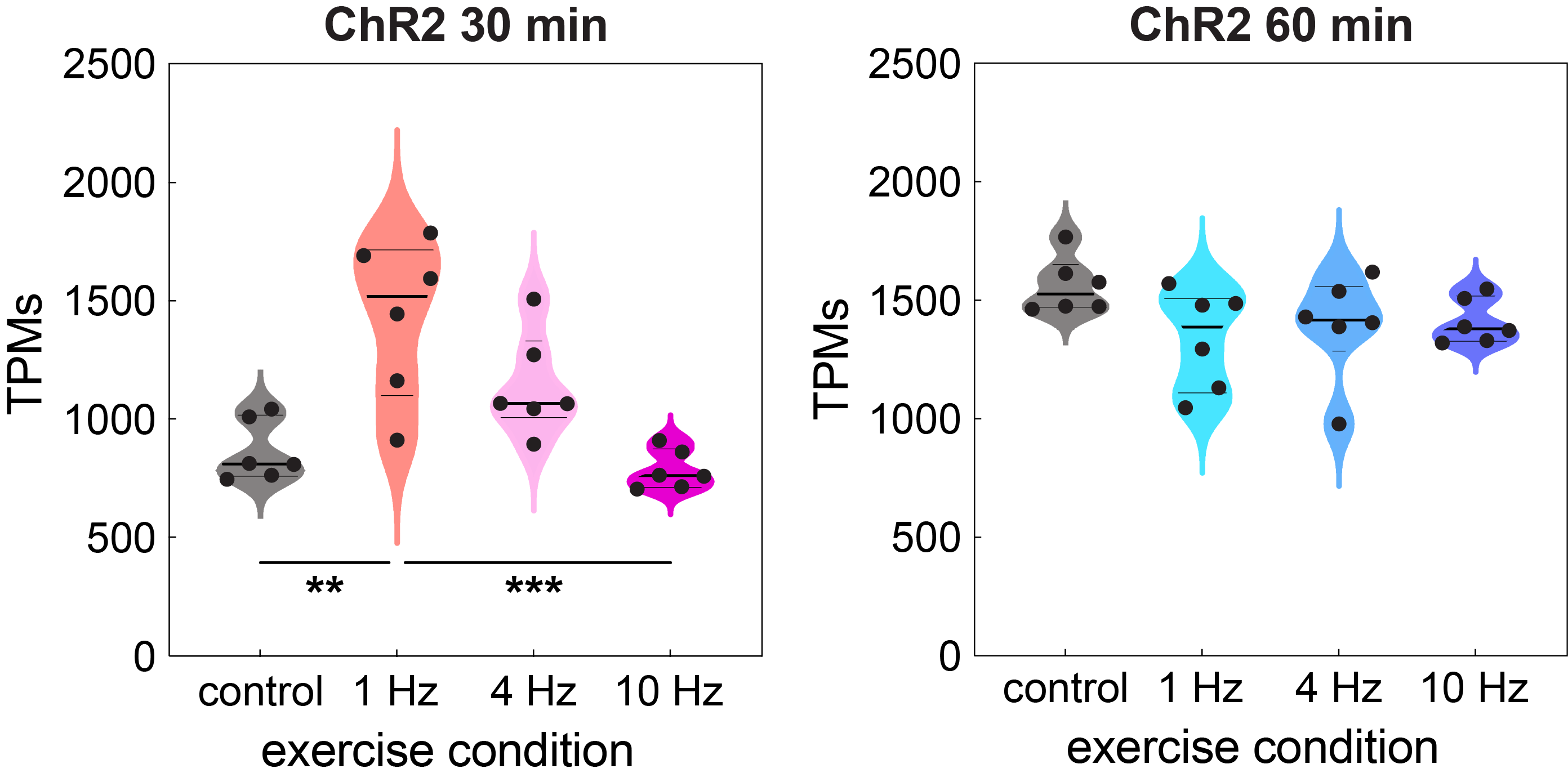


Figure S9: ChR2(H134R) gene expression in all conditions, quantified in transcripts per million (TPMs).


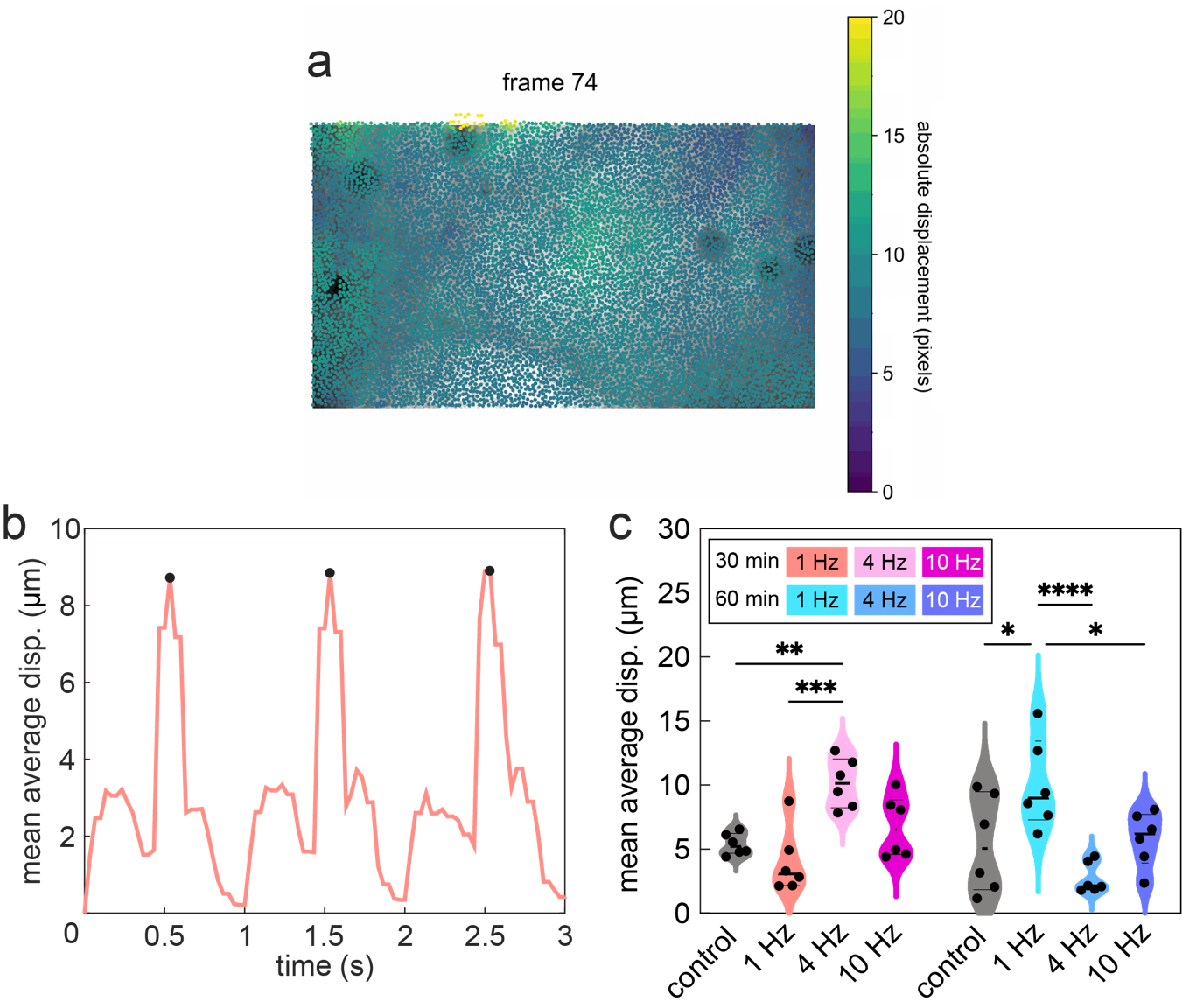


Figure S10: Twitch displacement comparison across experimental conditions on Differentiation Media (DM) Day 10. Contraction was triggered using electrical stimulation (10 V, 5 ms pulse width, 1 Hz). Data for a) and b) are taken from a biological replicate in the 1 Hz 30 min exercise training group. a) Frames from video recordings of muscle twitch are analyzed using strain tracking software (See Ref. 35). b) Mean average displacement values for recorded tissue area. Black dots at each peak are averaged to represent maximum displacements for each twitch cycle, then compiled with averages from other replicates to plot experimental condition displacements shown in c). All conditions were confirmed to be contractile on Day 10.

| **gene name** | **fiber type** | **description** |
| --- | --- | --- |
| Myh3 | embryonic |  |
| Myh8 | neonatal |  |
| Myh7 | type 1 | slow |
| Myh2 | type 2A | fast, oxidative |
| Myh1 | type 2X | fast, glycolytic |
| Myh4 | type 2B | fast, glycolytic |

Table S1: Descriptions of myosin heavy chain genes (Myh) and their associated fiber types.

**References**

1. Johnson A. error_ellipse. Published online July 23, 2015. Accessed September 13, 2025. https://www.mathworks.com/matlabcentral/fileexchange/4705-error_ellipse
